# Age-dependent induction of immunity and subsequent survival costs in males and females of a temperate damselfly

**DOI:** 10.1186/1472-6785-6-15

**Published:** 2006-11-07

**Authors:** Tonia Robb, Mark R Forbes

**Affiliations:** 1 Department of Ecology & Evolutionary Biology, 25 Willcocks Street, University of Toronto, Toronto, Ontario, M5S 3B2, Canada; 2 Department of Biology, 209 Nesbitt Building, Carleton University, Ottawa, Ontario, K1S 5B6, Canada

## Abstract

**Background:**

To understand variation in resistance to parasites within host populations, researchers have examined conditions under which immunity is induced and/or is costly. Both host sex and age have been found to influence immune expression and subsequently are likely factors influencing the costs of resistance. The purpose of this study was to examine immune expression and associated survival costs for two age groups (newly emerged and sexually mature individuals) of the damselfly, *Enallagma boreale *Selys. Survival was assessed for experimentally challenged and control damselflies, housed initially at 22°C and then subjected to low temperatures (15°C) associated with reduced foraging activity and food deprivation. Experimental conditions emulated natural local variation in bouts of good weather followed by inclement weather (successions of days with hourly mean temperatures around 15°C and/or rainy weather).

**Results:**

At least one of three immune traits was induced to higher levels for both newly emerged and mature *E. boreale *challenged by Lippopolysaccharide (LPS) relative to saline-injected controls, when housed at 22°C. The immune traits assayed included haemocyte concentration, Phenoloxidase activity and antibacterial activity and their induction varied among ages and between males and females. For matures, those injected with LPS had lowered survivorship compared to saline-injected controls that were housed initially at 22°C and subsequently at 15°C. Newly emerged LPS-injected damselflies did not show reduced survivorship relative to newly-emerged controls, despite showing immune induction.

**Conclusion:**

Reduced longevity following induction of immunity was observed for reproductively mature damselflies, but not for newly emerged damselflies. Costs of resistance depend only partly on the immune trait induced and more on the age (but not sex) of the host. In four years, we often observed bouts of inclement weather following good days and these bouts occurred primarily during the emergence periods, but also during the flight periods, of *E. boreale*. The duration of these bouts appear sufficient to compromise survival of mature damselflies that responded immunologically to LPS challenge. We further suggest the environmental conditions likely experienced by different ages of damselflies, following resistance expression, has influenced optimal immune investment by individuals in different age classes and the likelihood of detecting costs of resistance.

## Background

In natural populations, many species are host to one or more parasite species, where parasites are broadly defined to include viruses and bacteria. However, not all parasitic organisms elicit an immune response from individuals of a given host species [1,2]. Additionally, immune responses and the degree of resistance often vary among hosts, even when the same species of parasite is monitored in observational studies or used in experimental challenges [3-5]. Research has now focused on both the intrinsic and extrinsic factors that account for this variation in immune responses among hosts and the consequences of resistance to both host and parasite individuals and populations [5-7].

Evolutionary ecologists have adopted various approaches in attempting to explain within-population variation in immune responses and resistance expression. For example, researchers have examined the extent to which resistance is heritable and whether host responses are specific to the parasite strains used [8,9]. Other researchers have viewed the fitness benefits of immune defence as being traded off against other traits; an approach used in testing predictions of life-history theory [7,10]. Importantly, costs of resistance can include intrinsic costs of maintaining immune components in anticipation of parasitism and/or costs of induction i.e., initiating and activating an immune response. It is the latter costs that often are demonstrated for vertebrates and invertebrates in response to challenges from parasites or surrogates of parasitism (for invertebrates, insertion of a nylon filament or injection of Lipopolysaccharides in solution) [11,12].

The costs of resistance are expected to be context dependent, a problem only recently identified [13]. One study to examine context dependent resistance found that starved and immune challenged bumblebee workers had lower survivorship than either fed and challenged workers or starved workers that were not challenged [14]. That study and more recent work [15] has underscored the fact that success of parasites and their impact on their hosts, also should relate to environmentally relevant external factors.

Under natural conditions, insects are subjected to hours or days when foraging is restricted as a result of variable or inclement weather conditions. Hosts less able to acquire dietary resources may subsequently mount a less effective immune response [16,17]. Temperature also can play a key role in host response to parasitism [5]. Yet, little is known of the costs of resistance when temperatures, under which resistance is expressed, are not maintained. We expect when weather conditions are 'good' (resources are not limiting and temperatures are optimal) insects resist parasites. However, what happens if those insects are subjected to 'poor' conditions after allocating resources to resistance? Resistance costs may not be realised unless the host expressing resistance is subsequently challenged environmentally; further, these costs also may depend on the type of immunity induced and the magnitude of induction.

There have been several studies examining insect immunity; however, patterns of how insects respond to immune challenges are inconsistent and often relate to the immune traits assayed [18-22]. As a primary component of invertebrate immunity, circulating haemocytes are involved in recognition, phagocytosis and encapsulation of invading parasites and pathogens [23,24]. Activation of the pro-Phenoloxidase cascade to produce melanin is a key component of the invertebrate immune system (the production of melanin has both cytotoxic effects and antimicrobial properties [25]). In addition, anti-microbial peptides can be produced in response to an immune insult [25].

The main purpose of this study was to examine a direct fitness cost in relation to induction of an immune response(s) for adults of the temperate and early-emerging damselfly, *Enallagma boreale *Selys. To ensure our experiment included environmentally relevant conditions (e.g. periods of 'good' weather followed by successions of days of 'poor' weather), we first assessed the local variation in temperature and rainfall during the emergence and flight period of *E. boreale *for four previous emergence and flight periods (2001–2004). Both low temperatures and periods of heavy rainfall prevent foraging attempts for damselflies under natural conditions and were therefore considered 'poor' weather conditions [26]. Damselflies were immune challenged with a dose of immunogenic Lipopolysaccharide (LPS) in saline solution and allowed to respond at an environmentally relevant 'good' temperature. LPS or bacterial cell wall components are known to induce an immune response in several other insects without the pathogenic effect of the bacteria (e.g. [14]). To ensure LPS induced an immune response in *E. boreale*, we assessed immune traits of a subsample of LPS-injected and control (saline-injected) *E. boreale*. We assayed three key aspects of insect immunity: haemocyte concentration, Phenoloxidase (PO) activity and antibacterial activity. Our expectation was that at least one of these immune responses would be induced to higher levels when *E. boreale *were immune challenged. The remaining challenged damselflies were subsequently subjected to an environmentally relevant 'poor' temperature and food deprivation. To examine if costs of immune induction were realised when a responding individual is subsequently subjected to 'poor' environmental conditions, survivorship was assessed at cooler temperatures. Reduced adult longevity was expected at cooler temperatures and was expected to relate to the level of immune induction.

Sex differences in immunity are present, and can be understood by reference to natural or sexual selection on the sexes [10,27]. However, there is currently no general pattern of sex differences in immune investment by insects, as has been suggested for mammals [28]. Notwithstanding, host sex is an important factor to consider when assaying immune responses and the ultimate or evolutionary cost of resistance in anticipation of parasitism [29]. As with many insect species, male and female damselflies differ in life-history strategies: males appear to forage to obtain enough resources for mating activity whereas females forage to obtain greater resources for egg production and thereby gain weight during maturity [30]. Of course, the more important question is whether fitness relations to longevity differ for males or females leading to the expectation of higher immunity and longevity in either sex.

Costs of resistance and/or induction of immune defence also are expected to relate to the age of the host. Damselflies have two distinct adult stages, newly emerged (within 24 h of emergence) and reproductively mature [31]. Newly emerged damselflies must allocate resources to cuticular hardening as well as contend with parasites and pathogens. Enzyme pathways used in the production of melanin for immune defence are similar to those used in cuticular hardening [23,32]. Thus optimal investment in immunity may be limited by the maturation process. Further, based on observations from four previous years, newly emerged damselflies are expected to experience frequent and longer bouts of inclement weather (see methods). In comparison, reproductively mature adults have to contend with costs of reproduction as well as costs of defence if responding to challenge by parasites and/or pathogens. However, mature adults are expected to be most active later in the season when weather conditions should be more favourable (see methods). For another damselfly *Lestes virdis*, differences in immune parameters between newly emerged and mature damselflies were found and explained as age-related differences in life-history trade-offs [33]. The cost of immune induction also may be age-dependent reflecting the optimal patterns of energy allocation during maturation. For example, sex differences in immunity were evident for mature *Scathophaga stercaria *flies; however, this sex difference was not found for newly emerged flies [34]. This inconsistency between mature and newly emerged flies was explained as a result of sex-specific physiological requirements that were age-dependent.

As part of our main objective, we specifically compared *E. boreale *males and females of newly emerged and reproductively mature adults as we suspected both age and the sex would influence the type and magnitude of immune expression and subsequent costs of immune induction. Multiple measures of immunity ensured identification of differences in the specific immune trait expressed between males and females within each age category. Survivorship, as a measure of cost of resistance mediated by immune induction, was assessed for control and experimental individuals, which were either newly emerged or reproductively mature males or females.

## Results

### Immune Parameters

Immune parameters were not correlated for newly emerged (*r *ranged from 0.02–0.35, *p *values ranged from 0.10–0.92) or mature adults (*r *ranged from 0.008–0.35, *p *values ranged from 0.08–0.94) therefore each dependent variable was analyzed separately.

**Figure 1:**
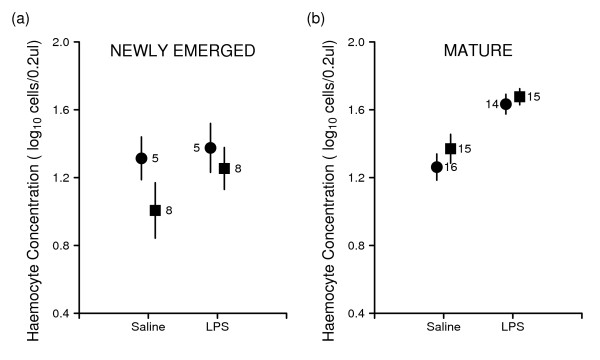
Mean haemocyte concentration (± 1 SE) for males (circle) and females (square) of (a) newly emerged and (b) mature *Enallagma boreale *12 h after injection of saline or LPS and held at 22°C. Numbers indicate sample size.

**Figure 2:**
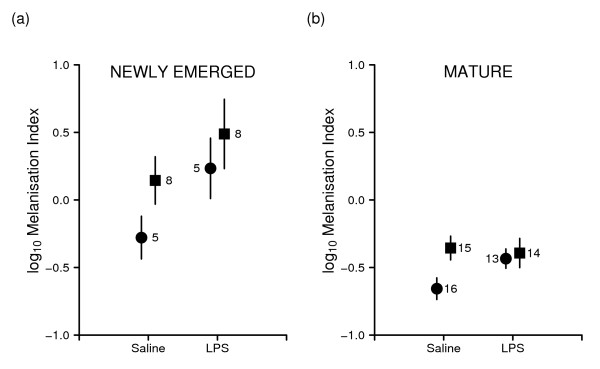
Mean melanisation index (± 1 SE) for males (circle) and females (square) of (a) newly emerged and (b) mature *Enallagma boreale *12 h after injection of saline or LPS and held at 22°C. Melanisation index values provide an indication of Phenoloxidase (PO) activity i.e. higher values indicates higher PO activity. Numbers indicate sample size.

**Figure 3:**
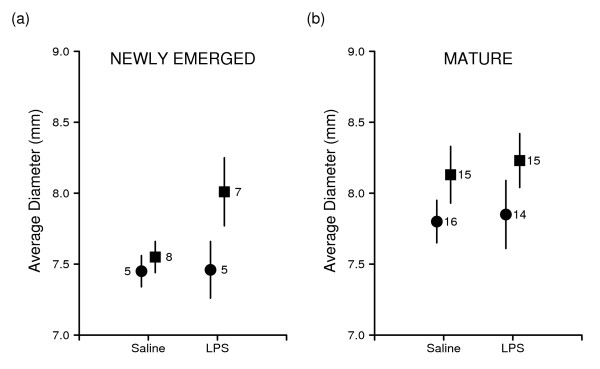
Mean average diameter of zones of antibacterial inhibition (± 1 SE) for males (circle) and females (square) of (a) newly emerged and (b) mature *Enallagma boreale *12 h after injection of saline or LPS and held at 22°C. Numbers indicate sample size.

Haemocyte concentration (cells/0.2 μl) did not differ between treatments or between sexes of newly emerged *E. boreale *(Figure 1a; sex *F*_1,21 _= 2.77, *p *= 0.11, treatment *F*_1,21 _= 1.07, *p *= 0.31, interaction *F*_1,20 _= 0.36, *p *= 0.55). However, both newly emerged males and females did have a higher melanisation index (MI; our measure of PO activity as detailed in the methods) when injected with LPS versus saline (Figure 2a; sex *F*_1,22 _= 1.59, *p *= 0.22, treatment *F*_1,22 _= 4.21, *p *= 0.05, interaction *F*_1,21 _= 0.16, *p *= 0.70). To assess antibacterial activity, several plates were seeded with bacteria (see methods). Controlling for the variability in bacterial growth between plates, there was a sex-by-treatment interaction when comparing antibacterial activity of LPS and saline injected newly emerged males and females (sex *F*_1,1 _= 352.83, *p *= 0.03, treatment *F*_1,1 _= 0.002, *p *= 0.97, interaction *F*_1,1 _= 4984.91, *p *= 0.009). Females and not males injected with LPS had heightened antibacterial activity (Figure 3a).

Mature males and females injected with LPS did have a greater haemocyte concentration than saline injected conspecifics (Figure 1b; sex *F*_1,57 _= 1.64, *p *= 0.21, treatment *F*_1,57 _= 24.07, *p *< 0.001, interaction *F*_1,56 _= 1.62, *p *= 0.21). The MI for both saline and LPS injected mature females was significantly greater than conspecific males, but there was no treatment effect (Figure 2b; sex *F*_1,54 _= 4.09, *p *= 0.04, treatment *F*_1,54 _= 1.03, *p *= 0.32, interaction *F*_1,53 _= 2.08, *p *= 0.16). Mature females had a stronger antibacterial activity than males when controlling for the variability between bacterial plates (Figure 3b; *F*_1,8 _= 5.15, *p *= 0.05). No difference in antibacterial activity between the treatments was observed for mature damselflies (Figure 3b; *F*_1,8 _= 0.09, *p *= 0.78, interaction *F*_1,8 _= 2.41, *p *= 0.16).

### Survivorship

**Figure 4:**
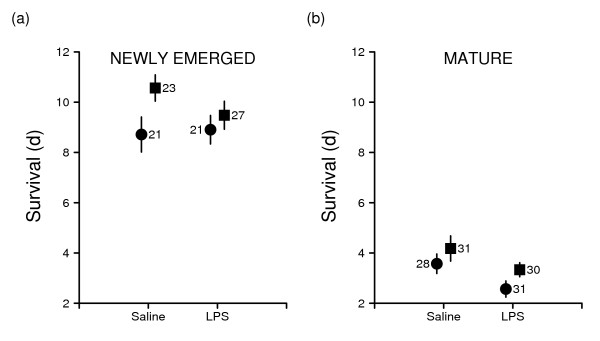
Mean days (± 1 SE) survived for males (circle) and females (square) of (a) newly emerged and (b) mature *Enallagma boreale *after injection of either saline or LPS and held at 15°C after an initial 24 h at 22°C to allow response to LPS. Numbers indicate sample size.

Newly emerged adult *E. boreale *injected with LPS did not have a significantly lower survivorship than conspecifics injected with saline (Figure 4a; ANOVA *F*_1,88 _= 0.72, *p *= 0.40). However, newly emerged females did survive longer than males in both treatments (Figure 4a; *F*_1,88 _= 3.95, *p *= 0.05, interaction *F*_1,88 _= 0.62, *p *= 0.43). Survivorship of mature *E. boreale *was lower in the LPS treatment compared to saline injected conspecifics (Figure 4b; ANOVA *F*_1,117 _= 5.81, *p *= 0.02). Similar to the newly emerged *E. boreale*, mature females lived longer than conspecific males in both treatments; however, the result only approached significance (Figure 4b; *F*_1,117 _= 3.54, *p *= 0.06, interaction *F*_1,117 _= 0.04, *p *= 0.83).

## Discussion

**Figure 5:**
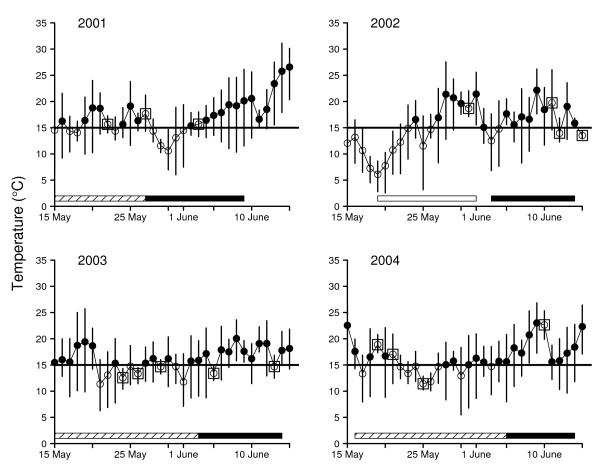
Mean hourly temperatures (± maximum and minimum temperatures for the foraging period 0400 h to 1900 h) during the emergence and approximate flight period of *Enallagma boreale *for the years 2001–2004 at Jack's Marsh. Closed circles represent good days of weather while open symbols represent poor weather. Bouts of poor weather were considered to be at least two consecutive days of poor weather. The line marked at 15°C indicates the cut off for our measure of poor weather and open squares indicate days with > 5 mm of rain, also indicating poor weather. The open bar represents the period of time when newly emerged individuals were observed and/or collected at Jack's Marsh. Open bar with hatch marks represents an estimated period of time when newly emerged individuals would likely have been observed at Jack's Marsh (reproductive maturity takes approximately 10–12 d to occur). The closed bar represents the period of time when mature adults were collected at Jack's Marsh.

A cost of immune induction in terms of adult longevity was evident for immune challenged mature *E. boreale *when subjected to low temperatures and food deprivation. There was approximately 25% reduction in adult survival (from on average 3.88 d to 2.94 d) when immune challenged damselflies were compared to saline injected controls. Two other studies have found similar results where upon activation of the immune system with LPS in adult bumblebees [14] and male field crickets [35], survivorship was lowered. Fellowes et al. [36] also found that larval *Drosophila *had a reduced competitive ability that likely leads to reduced survivorship when resistance was selected. Costs are not limited to just this specific immune challenge (LPS) and immune expression; upon induction of cellular encapsulation of a foreign body, *Tenebrio molitor *females had lowered survivorship compared to control females [37]. Our results suggest that under natural conditions, mature damselflies would pay the cost of resistance when faced with subsequently poor weather conditions. For this particular *E. boreale *population, there are many occasions when bouts of poor weather follow good weather (Figure 5), but these were more restricted to emergence periods. Importantly, resistance against parasitic mites (one of the most prevalent parasitic associations with adult Odonata [31]), when initiated, occurs at damselfly emergence [38]. In comparison, mature *E. boreale*, similar to other damselflies, ingest parasitic gregarines when feeding [39]. Although it is still unclear how or if damselflies respond to gregarines in the gut, our data suggest that future research should investigate whether mature damselflies responding to gregarines have lowered survivorship, when faced with inclement weather.

One question of interest is why survival of challenged newly emerged *E. boreale *was not significantly lower when compared to unchallenged controls. There is still some debate as to whether costs of resistance should occur, despite evidence in recent studies of trade-offs against other traits such as competitive ability or maturation rate [see [12,40]]. One explanation for our lack of finding a cost in newly-emerged adults is that newly emerged damselflies must contend with parasitic water mites in addition to repairing wounds inflicted by mites piercing the cuticle (melanin is often observed at the attachment of each mite; T. Robb personal observation). Secondary infection at the mite attachment points also may occur. For other insect hosts, risk of exposure as well as the type of challenge an individual is exposed to has explained how investment into immunity is optimised and subsequently how costs of resistance are minimised [41,42]. It is expected that newly emerged *E. boreale *with a high risk of parasitism have allocated more resources to potential immune responses than have mature reproductive *E. boreale*.

Another explanation concerns the nature of the cost of resistance. At emergence, damselflies must invest in cuticular hardening, a process that uses the same enzyme pathways (i.e. proPO cascade) as the production of melanin used in immune defence [23,32]. Thus, a more likely cost of resistance or in repairing wounds for newly emerged damselflies is longer times for cuticular hardening after eclosion. It is also important that newly emerged *E. boreale *responded to the immune challenge by increasing levels of PO activity and females responded with an increase in antibacterial activity; however, mature individuals responded to the LPS challenge with a significant increase in haemocyte concentration (Table 1). This difference in immune activation also may be partially responsible for our inability to find a cost of immune induction under environmentally relevant conditions for newly-emerged damselflies. Newly emerged damselflies also are more likely to encounter poor weather conditions, in particular for long periods of time in this study population (Figure 5). Responding to parasitism on good days does not appear to represent a cost for newly emerged *E. boreale *when inclement weather follows. In fact, under low temperature and reduced foraging conditions newly emerged *E. boreale *could live up to ten days at 15°C despite responding to an immune challenge (typical Zygoptera lifespan is up to 23 d [31]).

Both newly emerged and mature females did live longer than males in both treatment groups. A larger body size (particularly mass) could explain the greater female survivorship compared to male conspecifics for both newly emerged and mature *E. boreale*. For mature females, egg resorption also might occur to maintain longevity following resistance expression [43]. In comparison, immune challenged reproductive female *Tenebrio molitor *beetles suffered reduced longevity while maintaining fecundity [37]. It is expected that clutch size is less important to reproductive success of female damselflies than is the number of clutches over her lifetime [44] – this may not be the case for *T. molitor *females.

Sex differences in immune measures were evident where mature females had significantly greater levels of PO activity when challenged or not challenged compared to conspecific males. Both haemocytes and PO are involved in sclerotization or tanning of egg coverings (involved in the egg viability) in invertebrates [45,46]. Thus, heightened immune traits such as haemolymph PO activity in females may not be indicative of greater immunity, but rather a side-effect of a greater need for melanin. Differences in antibacterial activity were observed between the newly emerged sexes only in response to the immune challenge. However, no sex differences were observed between newly emerged *E. boreale *in our measures of PO activity or haemocyte concentration. Our results were similar to that found for the non-territorial damselfly, *Lestes viridis*, where newly emerged and mature damselflies demonstrated no difference in haemocyte totals, but where only PO activity was higher in mature females than conspecific males [33].

We did not find any evidence to suggest the cost of immune induction differed between males and females. This is a surprising result as previous work has indicated female immune investment is often greater than male investment [19,21,33,47]. Several hypotheses have been proposed to predict invertebrate sex differences in investment into immunity. Females are expected to invest in egg production whereas males must compete to fertilize eggs. Further, Rolff [48] suggested that assuming greater longevity will increase lifetime egg production; females will invest more into immunity compared to conspecific males. Alternatively, males downregulate or suppress immunity as a result of costly secondary sex ornaments and behaviour [49,50]. Recently, Mckean & Nunney [15] have suggested that sex differences in investment in resistance arise from male and female differential response to the environment: a topic of considerable relevance to the present study. They suggest the cost of resistance will depend not only on the investment in reproduction but will also relate to variation in fitness-limiting resource availability. Our data suggests that while allocation to immune parameters differed between mature males and females, there were no detectable differences in the costs of immune induction (survival) despite being subjected to similarly poor conditions (Table 1).

## Conclusion

The results suggest that costs of immune induction do not depend just on the type and magnitude of investment in immunity. We found that costs of immune induction in terms of longevity were evident for mature damselflies but not for newly emerged damselflies. The variation in weather conditions normally experienced by different aged individuals in this temperate damselfly, the type of immune expression, and when resistance against parasitism is typically mounted, are likely explanations for the age related differences in relation to experimental challenges that we observed. It is important that the environmental relevance of laboratory conditions are well founded for future studies on costs of immune induction in the laboratory.

## Methods

### Environmental Relevance

An important aspect of this study was to ensure that our methods of testing for costs of immune induction were environmentally relevant. A standard Stevenson screen at 1.5 m height with a Campbell Scientific 21X datalogger was operational near our collection site (Queen's University Biological Station) and was used to obtain temperature data. Temperature was recorded at five-second intervals, allowing us to examine mean hourly temperatures from 0400 h to 1900 h (representing sunrise to sunset or possible foraging times of *E. boreale *in our study region, *cf*. [51]). Mean hourly temperatures for each day were examined during the emergence period of tenerals as well as flight period of mature adults (approximately 15 May–15 June) for the four previous years (2001–2004) to the study. We assessed daily grand means of the mean hourly temperatures for each day over the emergence and flight periods of *E. boreale *in the study area (Figure 5). We also assessed rainfall during the daylight foraging times of *E. boreale*. Poor weather days were determined as the daily mean temperature less than or equal to 15°C and/or when greater than 5 mm of rain occurred indicating heavy rainfall and preventing periods of foraging.

We were interested in bouts of two or more successive days where daily grand mean temperatures were 15°C or lower and/or were characterized by heavy rainfall. We found 11 such bouts (the median duration was 3 d; inter-quartile range was 2–9 d). These bouts can be seen by examining Figure 5. Six of those bouts were during the emergence periods of *E. boreale *whilst three bouts were during the flight periods and the remaining two bouts bracketed the emergence and flight periods. The mean daily temperature on the days preceding each of the bouts ranged from 16–22°C (for the ten bouts where temperatures were recorded on the day previous). The maximum daily temperature during the daylight foraging times for those days ranged from 18–26°C. Based on these data, we chose 22°C to house LPS-injected and saline-injected damselflies for one day and 15°C to subsequently house damselflies following immune challenges versus control injections.

### Study Species

*Enallagma boreale *is a non-territorial damselfly with a mating system that can be defined as scramble mate competition [52]. *E. boreale *is found throughout eastern Ontario at beaver ponds and freshwater marshes [53]. Emergence of *E. boreale *usually begins at the end of May and mature adults are present through to mid to late June [53]. As with many dragonfly and damselfly species, *E. boreale *are subject to parasitism by larval water mites (Acari: Hydrachnida). During host emergence, the water mites transfer to newly eclosed adult damselflies and become parasitic [54]. The mites engorge during the host maturation period and return to the water during host reproduction. Host resistance to parasitism (melanotic encapsulation of the mite feeding resulting in death of the mite) will occur during the first 24–48 h after emergence [4].

### Collections

Male and female *E. boreale *were collected from Jack's Marsh near the Queen's Biology Station near Chaffeys lock Ontario, Canada (44° 34' N, 79° 15' W). Newly emerged damselflies were collected on a daily basis beginning 28 May through to the 1 June 2005 between 1000 h and 1200 h. For collections of mature adult damselflies, we collected the first individuals to be observed in tandem, ensuring males and females were reproductively mature and within a similar age group [31]. We collected reproductively mature damselflies on 6–8 and 10–11 June 2005 (no adults were observed in tandem on the 9 June between 1000 h and 1200 h). We attempted to collect equal numbers of damselflies each day. In total, 118 newly-emerged and 180 reproductively mature *E. boreale *were collected. Each day damselflies were brought back to the Queen's Biology Station (within 15 min of the collection site) in insect cages (30 cm × 30 cm × 40 cm) and placed in a cool (ca. 18°C) dark room for one hour. Individuals were weighed (± 0.001 g; Mettler AE100 Digital Scale) and placed in plastic cups (volume ca. 255 ml) covered with aluminium foil. Each plastic cup contained 2 ml of water under a metal mesh screen to prevent drowning of the damselfly and a wooden stick was placed in the cup to provide a perch. At this time, we enumerated parasitic water mites on each individual using a 20× loupe.

We assigned both newly emerged and mature damselflies used to assay immune parameters to treatment groups (LPS or saline injected) within a sex ensuring there were no differences in our measures of body size, wing length and body mass (*p *values range from 0.22 to 0.98). Before assessing survival, we also assigned males and females to treatments ensuring there was no difference in average mass or wing length between treatment groups within a sex (Table 2).

Parasitic water mites (*Arrenurus *spp.) observed on some damselflies could not be removed prior to experimentation without causing greater stress. Therefore, we ensured that the number of mites on newly emerged *E. boreale *did not differ between treatment groups for each sex prior to assays of immune parameters (zeros were included in analyses; males, *W *= 16, *p *= 0.99, n = 10; females, *W *= 28.5, *p *= 0.23, n = 16) and the survivorship experiment (Table 2). We also ensured that the number of mature *E. boreale *with mites was similar between treatment groups for each sex prior to assaying immune parameters and prior to conducting the survivorship experiment. However, we did not statistically compare mean mite intensity between treatments as the infection levels were low (see legend of Table 2). Of the 60 mature damselflies used to assess immune parameters 5 had mites-1 female with 14 mites and 1 male with 1 mite in the LPS treatment while 1 female with 18 mites and 2 males with 1 mite each were assigned to the saline treatment. Eight mature damselflies used in the survivorship experiment had mites out of the 120 in total or approximately 7% (Table 2). These individuals were not expected to sway analyses because they were few in number and their mite numbers were similar across treatments. Nonetheless, analyses were completed without these individuals, but did not change the overall outcome (see Results).

### Immune Parameters

To assess if activation of one or more immune parameters occurs at 22°C, 16 newly emerged females and 10 newly emerged males and 30 mature females and 30 mature males were examined. Damselflies used to assess immune induction were not used in survivorship experiments. To assess if an immune parameter was induced, we compared Lippolysaccharide (LPS)-injected individuals to saline-injected controls. Three assays were completed to assess immune activation for each individual and all were completed at the same time as the survivorship experiments.

Each individual assigned to LPS-injected group was injected through the ventral surface of the metaepimeron with 0.5 μl of 0.5 mg/ml LPS (Sigma) dissolved in ice-cold insect Ringers saline solution using a 32 gauge Hamilton Syringe (Hamilton Company). Individuals assigned to the saline treatment were similarly injected but with 0.5 μl of the insect Ringers saline solution alone. Immune activation was achieved through the injection of LPS and individuals were housed at 22°C for 12 h. We chose 22°C to ensure an immune response occurred within the 24 h. This temperature also was chosen for reasons detailed above and because it was an ideal temperature at which adult damselflies could be housed (T. Robb, personal observation). After 12 h at 22°C, we immediately collected haemolymph from the thorax of each injected damselfly by pushing 40 μl of ice cold sodium cacodylate buffer (0.01 M sodium cacodylate, 0.005 M calcium chloride, pH 7.4) through the thorax using a 26 gauge Hamilton Syringe. The haemolymph and buffer were collected in a 1.0 ml eppendorf held on ice. From this mixture of haemolymph and buffer we removed 10 μl for a haemocyte count and the remainder was frozen at -30°C to disrupt haemocytes for measure of PO activity and antibacterial activity. The number of haemocytes was determined using an improved Neubauer hemocytometer.

Phenoloxidase activity was determined by quantifying the conversion of L-3,4-dihydroxyphenylalanine (L-DOPA) to melanin catalyzed by PO [55]. Filter paper (Whatman No.52) cut in halves was soaked in 2 mg/ml L-DOPA (Sigma) in a sodium cacodylate buffer and 6 μl of the damselfly haemolymph solution was applied to the centre of the filter paper. Samples were kept for 30 min at room temperature and filter paper was kept moist with additional L-DOPA solution to allow excess substrate for the reaction to occur. Samples were then transferred to clean paper towel and allowed to dry. The end result was a circular melanised spot on the filter paper as a result of the PO present in the haemolymph solution. The PO activity was quantified by calculating a mean of the greyscale values for the melanised region. The addition of buffer alone resulted in no colour (melanisation) indicating melanin was produced as a result of the PO in the haemolymph. Multiple pieces of filter paper were scanned using a Cannon Scanner (CannoScan 9900F) set at 200 dpi, greyscale, 8 bits per colour, no automatic tone curve and high sharpness (60%). For each sample a mean greyscale pixel value of a circular region (364 pixels) at the visually estimated centre of the melanised spot was determined using UTHSCSA ImageTool program Version 3.0 [56]. A raw mean pixel value (RMPV) was calculated by subtracting the mean greyscale pixel value from 255; thus a value of 255 represented black and 0 represented white. To convert the RMPV values to a standardised measure of PO activity or the melanisation index (MI) a standard India ink solution (Faber-Castell Waterproof drawing ink no 4415) was used. The mean RMPVs for three aliquots at twelve dilution levels of the India ink solution was determined using the above methods (dilution levels range from undiluted to a dilution factor of 2^-12^, in decrements of powers of 2) and the best fit function of the curve (dilution versus RMPV) was calculated. The MI for each damselfly sample was computed as MI = e [(*RMPV*-*b*)/*m*] * 100% (where m was the slope of the best fit function and b was the intercept). For each individual the MI for three separate samples was determined and the mean MI was used for comparison of LPS injected versus saline injected controls.

Antibacterial peptides also are produced in response to infection and can be measured by testing haemolymph effectiveness at killing live bacteria (e.g. [14]). Antibacterial activity of the damselfly haemolymph was determined using a zone of inhibition assay against the bacteria *Arthrobacter globiformis*. Methods were completed similar to Moret & Schmid-Hempel [14]. Test plates (5 for newly emerged and 12 for mature adults) were made by adding 0.05 ml of *A. globiformis *(10^5 ^cells/ml) to 5 ml of nutrient agar and the plates were swirled to distribute bacteria evenly. Plates were left to settle at room temperature and then stored upside down at 4°C for up to two days prior to use. Six pieces of sterilised circular filter paper (6.29 mm in diameter) were placed on each test plate and 6 μl of the damselfly haemolymph mixture was pipetted on a single filter paper. Placement of samples on each plate and between plates was random to ensure each plate had samples from both treatments for each sex. On each plate, a negative control consisting of 6 μl of buffer alone was pipetted on a single filter paper. Plates were incubated upside down at 28°C for 24 h. The diameter of the clear zone around the filter paper was measured using digital callipers. The average of the minimum and maximum diameters was used for comparison. Bacteria growth was not inhibited by the buffer alone.

### Survivorship

Upon collection of newly emerged adult damselflies (50 females and 42 males), individuals were assigned to one of two treatment groups: saline-injected or LPS-injected. Injections and doses of LPS and saline were completed as above. After the injection, individuals were placed back in the plastic cups and held at 22°C (16:9 light:dark cycle, similar to that under natural conditions) for a 24-h time period to allow for the induction of the immune response.

Individuals were then placed into a 15°C incubator with the same light:dark cycle. This starvation and temperature regime should mimic 'real' occurrences of adverse weather and limited food availability. Damselflies were observed approximately every 12 h at which time if individuals were dead the date was recorded as well as wing length (± 0.01 mm; Mitutoyo digital callipers). Observations were completed until all individuals were dead. To determine survivorship differences of LPS-injected and saline injected mature adults, the same methods as outlined above were completed using reproductively mature *E. boreale *(61 females and 59 males). Differences in sample sizes between newly emerged and mature damselflies and between sexes occurred as a result of differences in number of individuals available at the field site during the collection periods.

### Statistical Analyses

All analyses were completed using R (version 2.1.1;[57]) and means are reported as ± 1 SE. Body mass, wing length, haemocyte concentration and MI were log transformed to meet the assumptions of normality. Analysis of antibacterial response was completed with plate as a random block effect to account for variation in bacteria between the plates. Two LPS injected mature damselflies (one male and one female) were removed from the MI analysis and one LPS injected newly emerged female damselfly was removed from the antibacterial analysis due to an error in the laboratory.

Separate analyses were completed for newly emerged and reproductively mature individuals and any non-significant interactions were removed for final analyses. We completed separate analyses because newly emerged and mature individuals were collected at different times of the season thus we could only explain the variation in survivorship and immune parameters within each age category and discuss if there were differences in explanatory variables.

## Authors' contributions

TR carried out the field collections and laboratory procedures and drafted the manuscript. MRF participated in the study design and coordination and helped to draft the manuscript. Both authors read and approved the final manuscript.

## Figures and Tables

**Table 1:** A summary of the significant results from the immune trait assays and the survivorship experiment. See text and Figures 1–4 for exact values and statistical tests.

	Result	
	Newly Emerged	Mature
	
Immune trait assayed		
Haemocyte concentration	NS	LPS > saline
MI	LPS > saline	females > males
Antibacterial activity	LPS > saline^1^	females > males
Survival	females > males	LPS < saline
		females > males^2^

^1^Sex by treatment interaction only females showed induction of antibacterial activity with LPS injection.

^2^*p *= 0.06

**Table 2:** Means ± 1 SE of mass, wing length and median number of mites on newly emerged and mature *Enallagma boreale *for saline injected and LPS injected groups used in the survivorship experiment.

		Mass (g)^1^		Wing Length (mm)^1^		Mites^2^	
		Saline	LPS	Saline	LPS	Saline	LPS
		
Newly	Males	0.328 ± 0.007	0.324 ± 0.007	12.87 ± 0.15	12.91 ± 0.11	7 (3 – 10)	4 (3 – 11)
Emerged	Females	0.350 ± 0.008	0.345 ± 0.008	13.81 ± 0.15	13.64 ± 0.16	4 (2 – 10)	5 (1 – 12)

Mature	Males	0.304 ± 0.007	0.313 ± 0.007	13.07 ± 0.11	13.14 ± 0.09	0 (0-0)	0 (0-0)
	Females	0.464 ± 0.014	0.469 ± 0.010	13.79 ± 0.12	13.74 ± 0.13	0 (0-0)	0 (0-0)

^1^No differences in mass or wing length were found between treatment groups within each age and sex category (Newly Emerged: Mass-males, *t*_40 _= -1.52, *p *= 0.14; females, *t*_48 _= -1.53, *p *= 0.13; Wing length-males, *t*_40 _= 0.27, *p *= 0.79; females, *t*_48 _= 0.83, *p *= 0.41; Mature: Mass-males, *t*_57 _= -0.91, *p *= 0.36; females, *t*_59 _= -0.44, *p *= 0.66; Wing length-males, *t*_57 _= -0.50, *p *= 0.62; females, *t*_59 _= 0.31, *p *= 0.76).

^2^No differences between the median number of parasitic water mites (interquartile ranges are indicated in brackets) were found for saline injected and LPS injected newly emerged *E. boreale *(zeros were included in analyses; males, *W *= 161, *p *= 0.16; females, *W *= 275.5, *p *= 0.63). Few (8) mature adults were found with mites and therefore no statistical tests were completed, although medians and interquartile ranges are reported. Two males that were saline-injected carried 2 and 27 mites, three males that were LPS-injected carried 4, 5 and 15 mites. One female that was saline-injected carried three mites and two LPS-injected females carried one and two mites.
